# Comparison of Prototype Transparent Mask, Opaque Mask, and No Mask on Speech Understanding in Noise

**DOI:** 10.3390/audiolres15040103

**Published:** 2025-08-11

**Authors:** Samuel R. Atcherson, Evan T. Finley, Jeanne Hahne

**Affiliations:** 1 Department of Audiology and Speech Pathology, University of Arkansas for Medical Sciences, University of Arkansas at Little Rock, Little Rock, AR 72205, USA; evan.finley@gmail.com; 2 Department of Otolaryngology—Head & Neck Surgery, University of Arkansas for Medical Sciences, Little Rock, AR 72205, USA; 3 FaceView Mask, LLC, Medford, OR 97504, USA; jeannehahne@gmail.com

**Keywords:** background noise, speech perception, face masks, transparent, hearing loss

## Abstract

**Background:** Face masks are used in healthcare for the prevention of the spread of disease; however, the recent COVID-19 pandemic raised awareness of the challenges of typical opaque masks that obscure nonverbal cues. In addition, various masks have been shown to attenuate speech above 1000 Hz, and lack of nonverbal cues exacerbates speech understanding in the presence of background noise. Transparent masks can help to overcome the loss of nonverbal cues, but they have greater attenuative effects on higher speech frequencies. This study evaluated a newer prototype transparent face mask redesigned from a version evaluated in a previous study. **Methods:** Thirty participants (10 with normal hearing, 10 with moderate hearing loss, and 10 with severe-to-profound hearing loss) were recruited. Selected lists from the Connected Speech Test (CST) were digitally recorded using male and female talkers and presented to listeners at 65 dB HL in 12 conditions against a background of 4-talker babble (+5 dB SNR): without a mask (auditory only and audiovisual), with an opaque mask (auditory only and audiovisual), and with a transparent mask (auditory only and audiovisual). **Results:** Listeners with normal hearing performed consistently well across all conditions. For listeners with hearing loss, speech was generally easier to understand with the male talker. Audiovisual conditions were better than auditory-only conditions, and No Mask and Transparent Mask conditions were better than Opaque Mask conditions. **Conclusions:** These findings continue to support the use of transparent masks to improve communication, minimize medical errors, and increase patient satisfaction.

## 1. Introduction

The COVID-19 pandemic highlighted communication challenges with face masks, which inspired numerous investigations in the years that followed. Prior to the onset of the COVID-19 pandemic, Mendel et al. [1] and Atcherson et al. [2] were among the first to explore the effect of masks on speech understanding in quiet and pre-recorded dental noise. Although Mendel et al. [1] did not see an effect of the mask in their normal hearing and moderate hearing loss listeners, they did report a slight decrease in performance in the presence of energetic masking dental office noise (as expected). Atcherson et al. [2] added a third group to include severe-to-profound listeners, used an energetic masking, 4-talker babble as the noise source, and introduced an early transparent mask prototype to compare with a standard, opaque surgical mask. They found that the noise and mask both disproportionately affected the listeners with hearing loss compared to the normal hearing listeners, that the audiovisual conditions yielded better results compared to the audio-only conditions, and that magnitude of improvement was greatest for the listeners with severe-to-profound hearing loss. These findings continue to hold true post-pandemic, and, not only with speech understanding, but also with speech production and acoustics, listening effort, quality of life, communication modality (e.g., spoken language vs. sign language), and socioemotional factors (see recent papers and reviews [3,4,5,6,7,8,9,10,11]). In terms of the acoustic effects of various masks, speech sounds are attenuated above 1000 Hz (more so for transparent masks), which alters higher-frequency components of speech and contributes to poorer speech understanding in both quiet and noisy situations (see, e.g., [12,13,14]). Because masks continue to be used in many healthcare settings, a strong case continues to be made for transparent masks even during non-pandemic periods [9]. Potential vulnerable patient groups include not only those with hearing loss but also young children, individuals with intellectual and cognitive disabilities, older adults, and individuals who speak languages or dialects different from the patient’s. These patients would undoubtedly benefit from having access to nonverbal cues by the mask wearer (e.g., the healthcare provider). Equally important is that transparent masks would be highly beneficial in working environments where noise levels interfere with speech understanding, even in individuals with normal hearing. In doing so, transparent masks can help to improve communication, minimize medical errors, and increase patient satisfaction.

Thus, the purpose of this study was to expand on the previous study conducted by Atcherson et al. [2] and to incorporate a newer transparent mask prototype design based on prior results and internal feedback. In contrast to the previous study, we also included both male and female talkers speaking sentences rather than just one gender, we created a new Opaque Mask audiovisual condition for comparison, and we decreased the 4-talker babble signal-to-noise ratio (SNR) +10 to +5 dB in efforts to create a more challenging scenario for the three groups based on hearing sensitivity.

## 2. Materials and Methods

### 2.1. Positionality Statement

As the first author (S.R.A.), I bring both insider and outsider perspectives to this study. As an insider, I have lived with hearing loss since early childhood, using hearing aids, cochlear implants, spoken English, and sign language, and have been an active member of the deaf and hard-of-hearing community for over four decades. I have personally experienced communication breakdowns when others wear opaque masks. As an outsider, I am an audiologist, health educator, and researcher. Since 2000, I have been involved with the Association of Medical Professionals with Hearing Losses (AMPHL), where communication challenges related to masks remain a persistent concern among members. These experiences led to the Mendel et al. [1], Atcherson et al. [2], and the current speech perception in noise studies involving masks.

I met the third author (J.H.), a nurse and transparent mask inventor, in 2011 at an AMPHL conference. Although she was not involved in the Atcherson et al. [2] study, she provided an early prototype of a transparent mask. J.H. later received a Small Business Technology Transfer (STTR) grant from the National Institute of Nursing Research (R41NR017124, 2019–2024), and a subaward was provided to me to conduct the current study using her latest prototype. The second author (E.T.F.) was a graduate research trainee funded by this subaward. Specific author contributions and conflict of interest statements are provided at the end of this paper.

### 2.2. Participants

**Figure 1:**
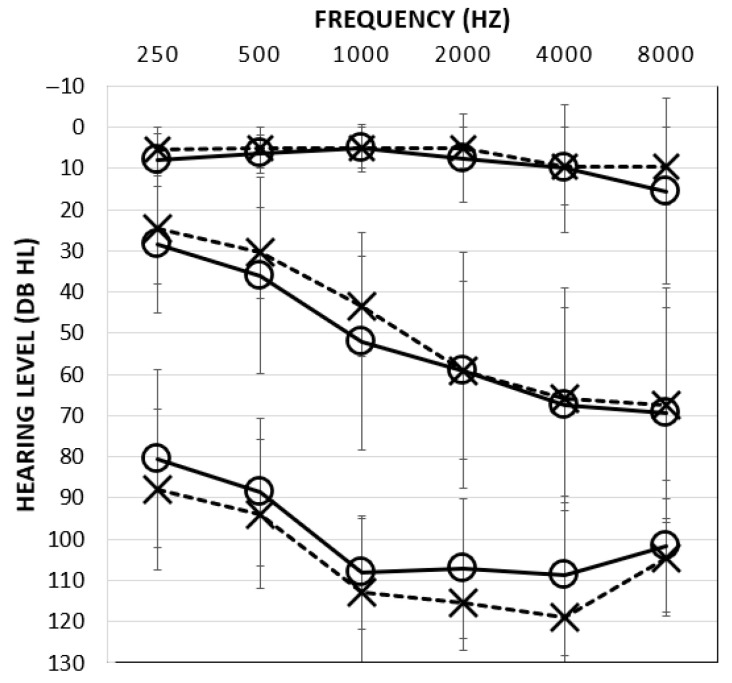
Mean air-conduction thresholds for NH (top), MOD (middle), and SEV (bottom) groups with errors reflecting ± 1 standard deviation bars. O = Right ear; X = Left ear.

Thirty adult participants between the ages of 18 and 73 years (x = 47.2, SD = 18.6) were recruited into one of three groups based on the three-frequency pure tone average hearing status of their better ear: normal hearing (NH; ≤25 dB HL), moderate to moderately severe hearing loss (MOD; 41–70 dB HL), and severe-to-profound hearing loss (SEV; <70 dB HL) (see Figure 1). Participants were 3 males and 7 females between the ages of 22 and 69 years ($\overset{-}{x}$ = 38.2, SD = 16.6) in the NH group, 6 males and 4 females between the ages of 24 and 77 years x = 57.3, SD = 18.1) in the MOD group, and 3 males and 7 females between the ages of 18 and 73 years xx = 45.6, SD = 17.8) in the SEV group. Demographic details, hearing devices, and mode of communication are summarized in Table 1.

All participants had normal middle ear function bilaterally as evidenced by normal tympanometric results (e.g., tympanometric peak pressure, ear canal volume, static admittance, and tympanometric width) using screening normative data from Roup et al. [15]. Participants were native communicators of American English, may use American Sign Language (ASL) in their responses, and had no major health issues. As the study utilized audiovisual materials, it was important for participants to have good visual capabilities with or without corrective lenses. A Snellen chart was used to verify good visual acuity by correctly reading letters representing 20/20 vision.

### 2.3. Masks

The masks used in this study were (1) a standard opaque medical mask with ASTM Level 3 protection (i.e., opaque mask) and (2) a newer transparent facemask prototype (i.e., FaceView™ mask) developed from the Small Business Technology Transfer (STTR) program grant awarded to J.H. by the National Institute of Nursing Health (R41NR017124) working towards a future N95 transparent medical respirator mask that meets regulatory requirements for use in healthcare. This new prototype was designed as an improvement over the previously evaluated prototype by Atcherson et al. [2], and, as a prototype, is not commercially available. The most notable difference between the current and previous transparent mask prototypes was changing from a bifold design to a trifold design. One internal criticism of the previous bifold design (see, e.g., [2]) was that the transparent film angled upward and creased during use, producing a visual light reflection and distraction during the study. The trifold design appeared to eliminate this issue with no crease and a forward-facing film.

### 2.4. Stimuli and Recording Instrumentation

With few exceptions to be reported here, stimuli (speech and noise) and instrumentation were similar to the Mendel et al. [1] and Atcherson et al. [2] studies. Differences in this study included a male and female talker recruited to reproduce passages from the Connected Speech Test (CST) [16,17] recorded at a distance of 1 m, under a number of conditions, and randomization of the conditions and some of the instrumentation used. Both talkers were White individuals with no facial hair and had a General American English dialect, providing consistent visual and auditory stimuli. In addition, both talkers had post-secondary education and graduate-level experiences with patients. To examine the effect of masks on speech understanding in noise performance in the three study groups, the CST stimuli were re-recorded using the male and female talkers for pre-randomized, pre-assigned list pairs. The 12 CST paired lists selected for this study were: 1–2, 3–4, 7–8, 9–10, 11–12, 13–14, 17–18, 19–20, 21–22, 29–30, 37–38, and 47–48. A professional teleprompter apparatus (Autocue, You Prompt YP12, Richmond, London, UK) mounted on a tripod was placed 1 m away from the speakers’ faces at 0° azimuth to record audiovisual conditions with and without the masks. All written CST passages were displayed through the teleprompter for the two speakers to read. Behind the teleprompter glass screen was a high-quality digital camera (Panasonic GC-GHP LumixG 4k, Panasonic Corporation; Kadoma, Osaka, Japan) mounted with 0° azimuth alignment. The speakers’ full faces and portions of their shoulders were recorded. A directional microphone (Rode VideoMic Pro Compact; Sydney, Australia) was coupled to the audio input of the digital camera but mounted on the teleprompter apparatus in front of the teleprompter screen. A sampling rate of 48 kHz and a 32-bit analog-to-digital converter were used to record the stimuli. To illuminate the speakers’ faces and eliminate shadows during the video recordings, two LED lights were placed at a 45° azimuth approximately 1 m from the speaker and, to eliminate the shadow cast by the two front LED lights, the last LED light was placed on a block facing upwards directly behind the speaker’s chair (ikan iLED312-v2-KIT, ikan Corporation; Houston, TX, USA). Following all accepted digital recordings, the 12 conditions were pre-randomized and combined into a single, full-length digital video along with a new 1000 Hz calibration tone matching the RMS level of all re-recorded CST passages combined. For background noise, we created a looped, 60 min sample of 4-talker babble (one male and three females) from the Bamford-Kowal-Bench Speech-in-Noise (BKB-SIN; Etymotic Research, Elkins Park, PA, USA) Test [18,19]. The final video was 16 min and 44 s long.

### 2.5. Study Procedures

Pure-tone thresholds were first measured for all participants at the octave frequencies from 250 to 8000 Hz using a diagnostic two-channel audiometer (GSI Audiostar Pro, Eden Prairie, MN, USA) with ER-3A insert earphones in a double-walled, acoustically shielded sound booth. Following audiometry, participants who typically used hearing devices were permitted to wear them for the speech perception in noise portion of the study. Prior to presentation of the CST stimuli, a 1000 Hz calibration tone was played to adjust the VU meter deflection of the audiometer to ‘0.’ The participants were then instructed in spoken English and/or ASL as follows: “You will hear several lists of topic-related sentences. Some of the sentences are presented so that you can see the talker on the video monitor. Some of the sentences are presented with the video screen blank. After you hear each sentence, please repeat it as clearly as you can. If you are unsure, please guess. Be sure to face forward and try to keep your head still”.

All stimuli were played from a desktop computer (Dell Optiplex 5050; Round Rock, TX, USA) outside the sound booth, and routed to both a full-color flat panel monitor (Dell 27 4k UltraSharp U2718Q; Round Rock, TX, USA) and loudspeaker (LifeLine LLF-50; Platteville, WI, USA) both of which were inside the sound booth. The 4-talker babble was played from a CD player (Sony Compact Disc Player CDP-C245; New York, NY, USA). In order to mix and deliver the speech from the CD and the noise through a common loudspeaker, both signals were routed individually through one of the two channels of the same audiometer. Speech and noise stimuli were presented with a +5 dB SNR (i.e., noise at 60 dB HL and speech at 65 dB HL) and calibrated with their calibration tones zeroed out on the VU meter. The participants were seated 1 m away from the monitor and loudspeaker at 0° azimuth.

**Figure 2:**
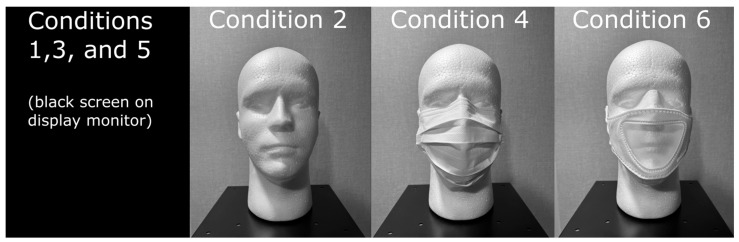
Examples of the conditions on a head model for the pre-recorded CST passages by the male and female talkers for a total of 12 conditions. Conditions 1 and 2 are NMA and NMAV, conditions 3 and 4 are OMA and OMAV, and conditions 5 and 6 are TMA and TMAV. Because conditions 1, 3, and 5 were audio-only, the display monitor showed a black screen.

All groups of research participants listened to four CST passages (2 male [M] paired passages, 2 female [F] paired passages, respectively) in each of the following audio-only [A] and audiovisual [AV] conditions (see Figure 2). Conditions 1, 3, and 5 were audio-only (A) with a black screen on the display monitor, while conditions 2, 4, and 6 were audiovisual (AV) with either the male or female talker donning one of the two mask types or no mask at all:

- Condition 1—NMA (No Mask Audio-only; Lists 19–20, 37–38).
- Condition 2—NMAV (No Mask Audiovisual; Lists 13–14, 47–48).
- Condition 3—OMA (Opaque Mask Audio-only; Lists 1–2, 21–22).
- Condition 4—OMAV (Opaque Mask Audiovisual; Lists 3–4, 17–18).
- Condition 5—TMA (Transparent Mask Audio-only; Lists 9–10, 11–12).
- Condition 6—TMAV (Transparent Mask Auditovisual; Lists 7–8, 29–30).

To aid in scoring for each session, audio and visual access to the participants sitting in the booth was achieved by using the previously used digital camera, routed to a recording monitor (Atomos Ninja Inferno 4k 60P; Cremorne, VIC, Australia) placed outside the sound booth. The recording monitor has an external hard drive that permits real-time audiovisual display and playback of all sessions. To enhance acoustic access to the participants and score their performance in real-time (as well as during playback), a lapel microphone was clipped near the participants’ mouths to allow clear response digital recording and scoring by examiners. Presentation of the test stimuli was paused to allow the participant time to repeat each item and the experimenters to score their responses. Testing sessions ranged from as little about 30 min to as long as 40 min, especially to accommodate participants who used ASL. Participants’ responses to the stimuli were scored as correct only if all key words were repeated correctly. Inter-judge scoring reliability of the listeners’ responses was calculated on 50% of the data from each group (i.e., NH, MOD, and SEV) to ensure accuracy in scoring the talk-back responses from the participants. The following formula was used: (agreements/[agreements + disagreements]) × 100%. Inter-judge scoring reliability was found to be 99%.

### 2.6. Data Analysis

Total root mean square (RMS) values and spectral analyses were compared for CST passages within the same stimulus experimental conditions (i.e., No Mask [NMA and NMAV], Opaque Mask [OMA and OMAV], and Transparent Mask [TMA and TMAV]). In order to calculate total RMS values, silent gaps between the sentences were deleted using Audacity audio software version 3.7.3 (Muse Group, San Francisco, CA, USA). Next, the total RMS values were determined for each of the three same stimulus experimental conditions (i.e., two list pairs per condition). For spectral analyses in logarithmic scale, the fast Fourier transformation (FFT) size was set to 65,536 (maximum), and the Blackman–Harris filter was applied. Next, FFT spectra were converted into 1/3rd octave bands for comparison across conditions and talkers.

For the speech perception in noise data, a three-way, mixed repeated measures ANOVA using IBM SPSS Statistics (Version 30) was conducted with Hearing Status (NH, MOD, and SEV) as the between-subjects factor and Talker (male, female) and Mask conditions (NMA, NMAV, OMA, OMAV, TMA, TMAV) as within-subjects factors. Although CST scores are typically reported in percentages, they were converted to rationalized arcsine units (RAU) [20] solely for statistical analysis to stabilize the error of variance and avoid ceiling and/or floor effects. In the event that the assumption of sphericity was not met, we applied Greenhouse–Geisser corrections when examining main effects and interactions. Alpha level was set at 0.05.

## 3. Results

### 3.1. Spectral Analysis of Stimuli

**Figure 3:**
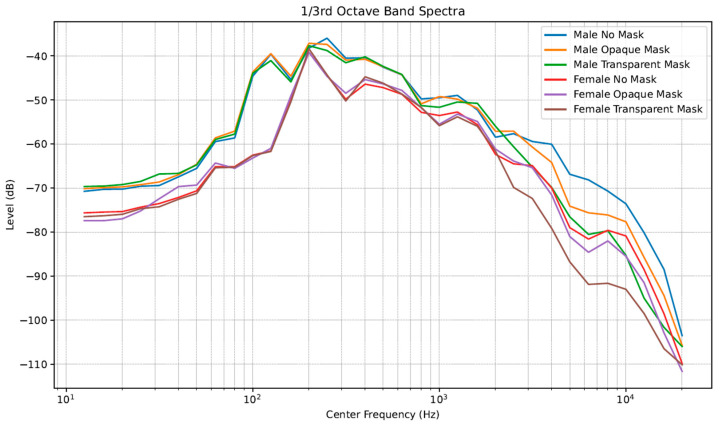
Spectral analyses of CST passages for male and female talkers and in No Mask, Opaque Mask, and Transparent Mask conditions.

Table 2 shows that the total RMS values progressively attenuated from No Mask to Opaque Mask and finally to Transparent Mask for the male talker, suggesting filtering by the two masks. However, for the female talker, the No Mask condition was slightly softer compared to the relatively equivalent Opaque and Transparent Mask conditions. In addition, the male talker was about 4 to 5 dB louder than the female talker. Figure 3 shows spectral changes differences between No Mask, Opaque Mask, and Transparent Mask conditions for the two talkers. Overall, spectra for the male talker revealed higher levels compared to the female talker. Between 1000 and 2000 Hz, it can be observed that the Opaque and Transparent Masks begin to show greater attenuation values compared to the No Mask condition, with the Transparent Mask producing the greatest attenuation after 2000 Hz for both male and female talkers.

### 3.2. Speech Perception in Noise Results

**Figure 4:**
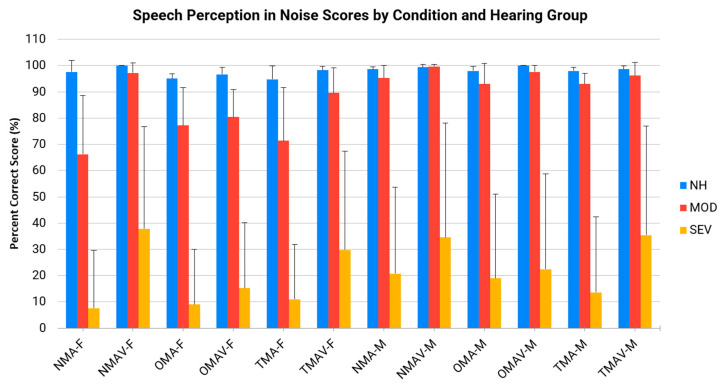
Mean percent correct performance for all three groups of participants (NH = Normal Hearing; MOD = Moderate; SEV = Severe-to-Profound) on the CST stimuli in the five experimental conditions (F = Female Talker; M = Male Talker; NMA = No Mask Auditory; NMAV = No Mask Audiovisual; TMA = Transparent Mask Auditory; TMAV = Transparent Mask Audiovisual; OMA = Opaque Mask Auditory; OMAV = Opaque Mask Audiovisual).

The primary purpose of this study was to evaluate the effect of talker (male and female), hearing status (NH, MOD, and SEV), and mask condition (No Mask, Surgical Mask, and Transparent Mask) on speech perception in noise performance. All CST results are presented in Figure 4.

A three-way, mixed repeated measures ANOVA revealed significant main effects for Hearing Status (F(2, 27) = 17.12, *p* < 0.001, η^2^_p_ = 0.56); Talker (F(1, 27) = 11.41, *p* = 0.002, η^2^_p_ = 0.30); and Mask condition (F(1.79, 48.45) = 17.86, *p* < 0.001, η^2^_p_ = 0.40). The Hearing Status × Mask condition interaction was significant (F(3.59, 48.45) = 5.25, *p* = 0.002, η^2^_p_ = 0.28), as was the Talker × Mask condition interaction (F(2.81, 75.90) = 5.24, *p* = 0.003, η^2^_p_ = 0.16). However, the three-way interaction (Hearing Status × Talker × Mask condition) was not significant (F(5.62, 75.90) = 1.55, *p* = 0.18). Post hoc Tukey HSD comparisons revealed that the NH group performed significantly better than both the MOD group (*p* = 0.003, *d* = 1.64) and the SEV group (*p* < 0.001, *d* = 2.59). The difference between the MOD and SEV groups was not statistically significant (*p* = 0.108), though the effect size was moderate (*d* = 0.94).

Simple main effects were assessed using one-way repeated measures ANOVAs separately for each Hearing Status group within a single Talker (male or female) across Mask condition. For any significant simple main effects, follow-up pairwise comparisons were conducted using paired-sample t-tests with False Discovery Rate (FDR) corrections [21,22]. For the male talker, the NH group did not significantly affect performance across Mask condition (F(5, 45) = 1.84, *p* = 0.124, η_p_^2^ = 0.17); however, there was a significant effect for the MOD (F(5, 45) = 2.77, *p* = 0.029, η_p_^2^ = 0.24) and SEV (F(5, 45) = 5.85, *p* < 0.0001, η_p_^2^ = 0.39) groups. These results suggest that neither Mask condition nor the +5 dB SNR had an effect on speech understanding for the NH group with the male talker. For the MOD group, pairwise comparisons showed that only the NMAV condition was statistically better than the NMA condition for the male talker, and no other comparisons reached significance. For the SEV group, pairwise comparisons showed that both the NMAV and TMAV conditions were statistically better than the NMA condition for the male talker, and no other comparisons reached significance.

For the female talker, there was a significant effect for the NH (F(5, 45) = 2.54, *p* = 0.041, η_p_^2^ = 0.22), MOD (F(5, 45) = 6.08, *p* = 0.0002, η_p_^2^ = 0.40) and SEV (F(5, 45) = 12.17, *p* < 0.001, η_p_^2^ = 0.58) groups. For the NH and MOD groups, pairwise comparisons showed that only the NMAV condition was statistically better than the NMA condition, and the TMAV condition was statistically better than the PMA condition for the female talker, and no other comparisons reached significance. For the SEV group, pairwise comparisons showed that both the NMAV and TMAV conditions were statistically better than the NMA condition, and the NMAV condition was statistically better than the PMA condition for the female talker, and no other comparisons reached significance.

Overall, the results demonstrate that Talker and Mask conditions interact to affect speech perception in noise, particularly for listeners with moderate and severe-to-profound hearing loss. Specifically, the male talker in this study was generally easier to understand compared to the female talker; audiovisual conditions were generally better than audio-only conditions; and the No Mask and Transparent Mask were generally better than the Opaque Mask.

## 4. Discussion

The purpose of this audiovisual speech perception in noise study was to compare a newer prototype transparent mask with an Opaque Mask and No Mask conditions in three groups of participants (NH, MOD, and SEV). Participants repeated audio-only and audiovisual CST stimuli, re-recorded and presented by a male or female talker against a 4-talker babble noise with an SNR of +5 dB. The total RMS levels showed progressive intensity degradations for the male talker from No Mask to Opaque Mask to Transparent Mask, but these differences were negligible (<1 dB), and for the female talker, No Mask was less intense than for both Opaque and Transparent Masks (also negligible at <0.5 dB). Compared to Mendel et al. [1] and Atcherson et al. [2], the negligible differences within individual talkers has two possible explanations: (1) standard, 3-ply surgical masks (i.e., opaque are amongst the least obstructive to acoustic energy [12,13,14] and (2) recordings were obtained 1 m from the talkers’ mouths as opposed to 10–15 cm which may effectively reduce the differences between masks. Total RMS levels were about 4–5 dB more intense for the male talker than for the female talker, which is well-supported by studies comparing the voice outputs between males and females [23,24]. Spectrally, the vocal output across frequencies illustrates additional differences not seen in total RMS levels (see Figure 3). Vocal intensities are similarly shown with the male talker being more intense than the female talker in all conditions, and there is the well-known attenuation effect of masks after 1000 Hz [12,13,14], more so for the Transparent Mask compared to the Opaque Mask. These acoustic effects can help to explain the speech perception in noise results as follows.

Participants with NH performed well (86% to 100%) in all conditions, regardless of Talker and Mask Type, and in the presence of noise. This finding is in agreement with Mendel et al. [1] and Atcherson et al. [2], who also used the CST passage materials at a fixed SNR. For the MOD and SEV hearing loss groups, averaged CST scores reflected differences in hearing loss sensitivity (SEV < MOD) and were always higher for the audiovisual conditions compared to the audio-only conditions. In addition, the use of +5 dB SNR with the 4-talker babble resulted in slightly poorer averaged CST scores in this study compared to the results under a +10 dB SNR in Atcherson et al. [2] for all groups, but proportionally worse for the two hearing loss groups. Comparing male vs. female talker conditions, averaged CST scores were almost always higher for the male talker than for the female talker. In only one paired contrast, the averaged CST scores were higher for the female talker in the No Mask audiovisual condition. Overall, these findings are expected and corroborated in other studies, even with other non-passage speech in noise materials [25,26].

Comparable to the findings in Atcherson et al. [2], there were functional differences in the use of hearing devices and communication modalities among the two hearing loss groups. Whereas their unaided audiogram results show clear differences in hearing sensitivity by group, functional variability resulted in wide variability of the CST scores in those two hearing loss groups. Though the NH group clearly outperformed the MOD and SEV groups overall, the lack of sufficient study power rendered the MOD and SEV groups insignificant from each other due to large standard deviations and overlap in scores. In both the MOD and SEV groups, younger adults were more likely to outperform older adults, and for the SEV group, cochlear implant users generally outperformed hearing aid users. While participants in the MOD hearing loss group were all spoken English users (i.e., oral English) and mostly bilateral hearing device users, participants in the SEV hearing loss group were more diverse. Six of 10 participants in the SEV group had CST scores no higher than 14% correct, suggesting that having hearing loss does not equate to better speechreading (audiovisual) or lipreading (visual only) abilities, at least in talkers unfamiliar to the listeners. As the study used clear speech under optimal listening and lighting conditions, the NH group performed well based on audition alone, even if visual cues were provided. However, for the MOD and SEV groups, the results were drastically different, with a study limitation of only one male and one female talker whose unique articulatory patterns may have negatively influenced the results for those who are much more visually dependent on speechreading (audiovisual) or lipreading (visual only).

There are several limitations to the current study. First, the demographics of the participant sample are influenced by variability of age within groups, and the subset of individuals with severe-to-profound hearing loss who were not able to take advantage of any auditory cues and could not lipread. Research has consistently demonstrated that speech perception in noise declines with age (e.g., [27]). Participants who could not take advantage of any auditory cues undoubtedly increased variability and consequently skewed the data towards poorer scores. Recruiting a more homogeneous group of severe-to-profound hearing loss with functional hearing may have been a better approach. Second, although it has been demonstrated that male voices tend to be more intense than female voices, the results may differ if the intensity differences were reduced (normalized) between talkers, leaving spectral differences to explain any differences. Finally, this speech perception in noise study was conducted under idealized conditions in an acoustically treated sound booth under adequate lighting, which always calls into question the potential ecological validity of the results. Specifically, can these results be translated to the real world given the variability of talkers, background noise, communicated context, etc.? Thus, the results of this study are potentially limited to the idealized setting in a laboratory environment.

Though this study only investigated the effect of masks on speech perception in noise, masks can have positive and negative effects on nonverbal cues and socioemotional processing. For example, both opaque and transparent masks can have detrimental effects on sign language comprehension and socioemotional processing by modulating some metacognitive and linguistic effects [6]. Here, even transparent masks can require greater mental effort and decrease confidence in understanding, even with sign language comprehension. The participants who used sign language in this study were greatly disadvantaged and were not able to take advantage of their native language. In terms of socioemotional processing, McCrackin et al. [28] found that opaque masks can negatively impact affective theory of mind (i.e., the ability to understand and predict the emotions and feelings of others) as well as affective empathy. Conversely, transparent masks were shown to restore affective theory of mind, but not affective empathy.

## 5. Conclusions

Although face masks remain a fixture in healthcare long after the height of a pandemic, the case for transparent alternatives is just as strong in routine clinical practice (see [9]). It is clear that having audiovisual access to speech and other nonverbal cues, whether with transparent masks or no masks, can be helpful to overall human interactions. Several patient groups—those with hearing loss, young children, people with cognitive or intellectual disabilities, older adults, and individuals speaking unfamiliar languages or dialects—rely heavily on visual cues from their caregivers [29,30]. Likewise, transparent masks can be invaluable in noisy environments where even people with normal hearing struggle to understand speech. By restoring access to lip movements and facial expressions, these masks not only enhance communication but also help prevent medical errors and elevate patient satisfaction. The prototypical transparent mask in this study presents a promising step towards the development of an N95-style transparent medical respirator mask for use in healthcare. Specifically, the transparent mask in this study did not appear to substantially influence speech in noise understanding within the different groups. Instead, the transparent mask did appear to provide improvements in audiovisual or visual-only access to lipreading and other nonverbal cues for those with hearing loss.

## Figures and Tables

**Table 1:** Characteristics of the Participants.

Participant	Group	Age	Gender	Amplification	Modality
1	NH	22	F	N/A	Oral (English)
2	NH	24	M	N/A	Oral (English)
3	NH	36	F	N/A	Oral (English)
4	NH	23	F	N/A	Oral (English)
5	NH	29	F	N/A	Oral (English)
6	NH	52	M	N/A	Oral (English)
7	NH	44	F	N/A	Oral (English)
8	NH	29	F	N/A	Oral (English)
9	NH	59	F	N/A	Oral (English)
10	NH	69	M	N/A	Oral (English)
11	MOD	35	F	1 CI; 1 HA	Oral (English)
12	MOD	24	M	2 HA	Oral (English)
13	MOD	70	F	2 HA	Oral (English)
14	MOD	77	M	2 HA	Oral (English)
15	MOD	46	M	2 HA	Oral (English)
16	MOD	67	F	2 HA	Oral (English)
17	MOD	68	M	2 HA	Oral (English)
18	MOD	46	F	N/A	Oral (English)
19	MOD	71	M	2 HA	Oral (English)
20	MOD	69	M	2 HA	Oral (English)
21	SEV	43	M	2 CI	Oral (English)
22	SEV	52	F	2 HA	SimCom
23	SEV	73	M	N/A	ASL
24	SEV	39	M	2 CI	Oral (English)
25	SEV	48	F	2 HA	SimCom
26	SEV	46	F	1 HA	Oral (English)
27	SEV	68	F	1 HA	SimCom
28	SEV	50	F	1 CI; 1 HA	Oral (English)
29	SEV	19	F	N/A	ASL
30	SEV	18	F	1 HA	ASL

Notes: ASL = American Sign Language; CI = cochlear implant; HA = hearing aid; N/A = not applicable or none; SimCom = simultaneous ASL and Oral (English).

**Table 2:** Total RMS values in dB for each experimental condition, talker, and difference between talkers.

Experimental Conditions	Male Talker	Female Talker	Difference
No Mask	−18.52	−23.85	5.33
Opaque Mask	−18.75	−23.48	4.72
Transparent Mask	−19.33	−23.45	4.12

## Data Availability

The original contributions presented in this study are included in the article. Further inquiries can be directed to the corresponding author.

## Abbreviations

The following abbreviations are used in this manuscript:

ANOVA	Analysis of Variance
ASL	American Sign Language
CI	Cochlear Implant
CST	Connected Speech Test
FFT	Fast Fourier Transformation
HA	Hearing Aid
NH	Normal Hearing Group
NMA	No Mask Audio-Only Condition
NMAV	No Mask Audiovisual Condition
OMA	Opaque Mask Audio-Only Condition
OMAV	Opaque Mask Audiovisual Condition
MOD	Moderate Hearing Loss Group
RAU	Rationalized Arcsine Units
RMS	Root Mean Square
SEV	Severe-to-Profound Hearing Loss Group
SNR	Signal-to-Noise Ratio
TMA	Transparent Mask Audio-Only Condition
TMAV	Transparent Mask Audiovisual Condition

## References

[1] Mendel L.L., Gardino J.A., Atcherson S.R. (2008). Speech understanding using surgical masks: A problem in health care?. J. Am. Acad. Audiol..

[2] Atcherson S.R., Mendel L.L., Baltimore W.J., Patro C., Lee S., Pousson M., Spann M.J. (2017). The Effect of Conventional and Transparent Surgical Masks on Speech Understanding in Individuals with and without Hearing Loss. J. Am. Acad. Audiol..

[3] Carroll S.M., Atcherson S.R. (2023). Living in a Limited World: Experience of Lipreaders When Society Is Masked. J. Psychosoc. Nurs. Ment. Health Serv..

[4] Badh G., Knowles T. (2023). Acoustic and perceptual impact of face masks on speech: A scoping review. PLoS ONE.

[5] Francis R., Leavitt M., McLelland C., Hamilton D.F. (2024). The influence of facemasks on communication in healthcare settings: A systematic review. Disabil. Rehabil..

[6] Giovanelli E., Gianfreda G., Gessa E., Valzolgher C., Lamano L., Lucioli T., Tomasuolo E., Rinaldi P., Pavani F. (2023). The effect of face masks on sign language comprehension: Performance and metacognitive dimensions. Conscious. Cogn..

[7] Grassi J., Oliveira I.B., Chiriboga L.F., Maia A.A., Attianezi M., Almeida A.N.P. (2023). Effects on communication due to face mask use: An integrative review. Rev. Bras. Enferm..

[8] Jackson I.R., Perugia E., Stone M.A., Saunders G.H. (2024). The impact of face coverings on audio-visual contributions to communication with conversational speech. Cogn. Res. Princ. Implic..

[9] McCrackin S.D., Ristic J. (2024). Improving masked communication: The case for transparent masks. Front. Commun..

[10] Rahne T., Fröhlich L., Plontke S., Wagner L. (2021). Influence of surgical and N95 face masks on speech perception and listening effort in noise. PLoS ONE.

[11] Saunders G.H., Jackson I.R., Visram A.S. (2021). Impacts of face coverings on communication: An indirect impact of COVID-19. Int. J. Audiol..

[12] Atcherson S.R., McDowell B.R., Howard M.P. (2021). Acoustic effects of non-transparent and transparent face coverings. J. Acoust. Soc. Am..

[13] Corey R.M., Jones U., Singer A.C. (2020). Acoustic effects of medical, cloth, and transparent face masks on speech signals. J. Acoust. Soc. Am..

[14] Goldin A., Weinstein B.E., Shiman N. (2020). How do medical masks degrade speech perception?. Hear. Rev..

[15] Roup C.M., Wiley T.L., Safady S.H., Stoppenbach D.T. (1998). Tympanometric Screening Norms for Adults. Am. J. Audiol..

[16] Cox R.M., Alexander G.C., Gilmore C. (1987). Development of the Connected Speech Test (CST). Ear Hear..

[17] Cox R.M., Alexander G.C., Gilmore C., Pusakulich K.M. (1988). Use of the Connected Speech Test (CST) with hearing-impaired listeners. Ear Hear..

[18] Bench J., Kowal A., Bamford J. (1979). The BKB (Bamford-Kowal-Bench) sentence lists for partially-hearing children. Br. J. Audiol..

[19] Niquette P., Arcaroli J., Revit L., Parkinson A., Staller S., Skinner M., Killion M. Development of the BKB-SIN Test. Proceedings of the Annual Meeting of the American Auditory Society.

[20] Studebaker G.A. (1985). A “rationalized” arcsine transform. J. Speech Hear. Res..

[21] Benjamini Y., Hochberg Y. (1995). Controlling the false discovery rate: A practical and powerful approach to multiple testing. J. Roy. Stat. Soc. Ser. B.

[22] Noble W.S. (2009). How does multiple testing correction work?. Nat. Biotechnol..

[23] Gelfer M.P., Young S.R. (1997). Comparisons of intensity measures and their stability in male and female speakers. J. Voice.

[24] Zhang Z. (2021). Contribution of laryngeal size to differences between male and female voice production. J. Acoust. Soc. Am..

[25] Mendel L.L., Pousson M.A., Shukla B., Sander K., Larson B. (2022). Listening Effort and Speech Perception Performance Using Different Facemasks. J. Speech Lang. Hear. Res..

[26] Thibodeau L.M., Thibodeau-Nielsen R.B., Tran C.M.Q., Jacob R.T.S. (2021). Communicating During COVID-19: The Effect of Transparent Masks for Speech Recognition in Noise. Ear Hear..

[27] Rahne T., Wagner T.M., Kopsch A.C., Plontke S.K., Wagner L. (2023). Influence of Age on Speech Recognition in Noise and Hearing Effort in Listeners with Age-Related Hearing Loss. J. Clin. Med..

[28] McCrackin S.D., Provencher S., Mendell E., Ristic J. (2022). Transparent masks reduce the negative impact of opaque masks on understanding emotional states but not on sharing them. Cogn. Res. Princ. Implic..

[29] Pavlova M.A., Sokolov A.A. (2022). Reading Covered Faces. Cereb. Cortex..

[30] Schneider J., Sandoz V., Equey L., Williams-Smith J., Horsch A., Bickle Graz M. (2022). The Role of Face Masks in the Recognition of Emotions by Preschool Children. JAMA Pediatr..

